# The Cytoprotective Effect of *Petalonia binghamiae* Methanol Extract against Oxidative Stress in C2C12 Myoblasts: Mediation by Upregulation of Heme Oxygenase-1 and Nuclear Factor-Erythroid 2 Related Factor 2

**DOI:** 10.3390/md13052666

**Published:** 2015-04-29

**Authors:** Ji Sook Kang, Il-Whan Choi, Min Ho Han, Dae-Sung Lee, Gi-Young Kim, Hye Jin Hwang, Byung Woo Kim, Cheol Min Kim, Young Hyun Yoo, Yung Hyun Choi

**Affiliations:** 1 Blue-Bio Industry RIC and Anti-Aging Research Center, Dongeui University, Busan 614-714, Korea; E-Mails: 13839@deu.ac.kr (J.S.K.); lab301@nate.com (H.J.H.); bwkim@deu.ac.kr (B.W.K.); 2 Department of Microbiology, College of Medicine, Inje University, Busan 608-756, Korea; E-Mail: cihima@inje.ac.kr; 3 Marine Biodiversity Institute of Korea, Seocheon 325-902, Korea; E-Mails: alsgh0615@lycos.co.kr (M.H.H.); lds8270@hanmail.net (D.S.L.); 4 Laboratory of Immunobiology, Department of Marine Life Sciences, Jeju National University, Jeju 690-756, Korea; E-Mail: immunkim@cheju.ac.kr; 5 Department of Food and Nutrition, College of Natural Sciences & Human Ecology, Dongeui University, Busan 614-714, Korea; 6 Department of Life Science and Biotechnology, College of Natural Sciences & Human Ecology, Dongeui University, Busan 614-714, Korea; 7 Department of Biochemistry, Busan National University College of Medicine, Yangsan 626-870, Korea; E-Mail: kimcm@pusan.ac.kr; 8 Department of Anatomy and Cell Biology, Dong-A University College of Medicine & Mitochondria Hub Regulation Center, Busan 602-714, Korea; 9 Department of Biochemistry, Dongeui University College of Korean Medicine, Busan 614-052, Korea

**Keywords:** *Petalonia binghamiae*, oxidative stress, ROS, DNA damage, Nrf2/HO-1

## Abstract

This study was designed to examine the protective effects of the marine brown algae *Petalonia binghamiae* against oxidative stress-induced cellular damage and to elucidate the underlying mechanisms. *P. binghamiae* methanol extract (PBME) prevented hydrogen peroxide (H_2_O_2_)-induced growth inhibition and exhibited scavenging activity against intracellular reactive oxygen species (ROS) induced by H_2_O_2_ in mouse-derived C2C12 myoblasts. PBME also significantly attenuated H_2_O_2_-induced comet tail formation in a comet assay, histone γH2A.X phosphorylation, and annexin V-positive cells, suggesting that PBME prevented H_2_O_2_-induced cellular DNA damage and apoptotic cell death. Furthermore, PBME increased the levels of heme oxygenase-1 (HO-1), a potent antioxidant enzyme, associated with the induction of nuclear factor-erythroid 2 related factor 2 (Nrf2). However, zinc protoporphyrin IX, a HO-1 competitive inhibitor, significantly abolished the protective effects of PBME on H_2_O_2_-induced ROS generation, growth inhibition, and apoptosis. Collectively, these results demonstrate that PBME augments the antioxidant defense capacity through activation of the Nrf2/HO-1 pathway.

## 1. Introduction

Oxidative stress resulting from a disturbance in the balance between the production of reactive oxygen species (ROS) and antioxidant defenses is the pathological basis of many chronic diseases. Mitochondria are multifunctional organelles that not only serve as cellular energy stores but are also actively involved in several cellular stress responses, including apoptosis. In addition, mitochondria themselves are also continuously challenged by stresses such as ROS [1]. Under normal physiological conditions, ROS are scavenged by the cellular antioxidant defense system. Some ROS act as cellular messengers in redox signaling. However, excessive production of ROS causes destructive and irreversible damage to all cellular components, including nucleic acids, proteins, and lipids [1,2]. The induction of antioxidant enzymes is one of the most important determinants of cytoprotective effects against oxidative stress-related diseases.

Previous studies have shown that the response of an inducible heme-degrading enzyme heme oxygenase-1 (HO-1), which is an antioxidant enzyme, to a wide range of cellular stresses exhibits adaptive responses to oxidative stress including skeletal muscle cells [3]. Therefore, targeted induction of this enzyme may be considered an important therapeutic strategy for protection against oxidative damage. Nuclear factor erythroid 2-related factor 2 (Nrf2) is a major transcription factor of HO-1. It acts as a master cellular sensor for oxidative stress and represents the primary response to changes in the cellular redox state [4,5,6]. Therefore, Nrf2 is considered a key target of antioxidant enzyme inducers in the primary defense mechanism against ROS: the conversion of highly toxic ROS to less reactive and less damaging forms.

Marine algae are rich sources of minerals, vitamins, and dietary fibers, and they have been used in medicinal herbs in East Asia for centuries [7,8]. Recent attention has focused on extracts, fractions, and single compounds derived from marine algae to develop new drugs and healthy foods, including *Petalonia binghamiae* (J. Agaradh) Vinogradova, a perennial brown alga (Phaeophyta), which belongs to the Alariaceae family and is distributed in the middle Pacific coast around Korea and Japan. Previous studies have shown that this edible brown alga inhibited adipocyte differentiation [9]. It was also shown that this alga has antiobesity [10] and antidiabetic properties [11]. Although the antioxidant potential of *P. binghamiae* has been reported [12], no study has been conducted to examine the protective capacity of *P. binghamiae* against oxidative stress. In the present study, we examined the ability of *P. binghamiae* methanol extract (PBME) to protect cells from hydrogen peroxide (H_2_O_2_)-induced cell damage and elucidated the mechanism underlying these protective effects in a mouse-derived C2C12 myoblast model.

## 2. Results and Discussion

### 2.1. PBME Reduces H_2_O_2_-Induced C2C12 Cytotoxicity

**Figure 1:**
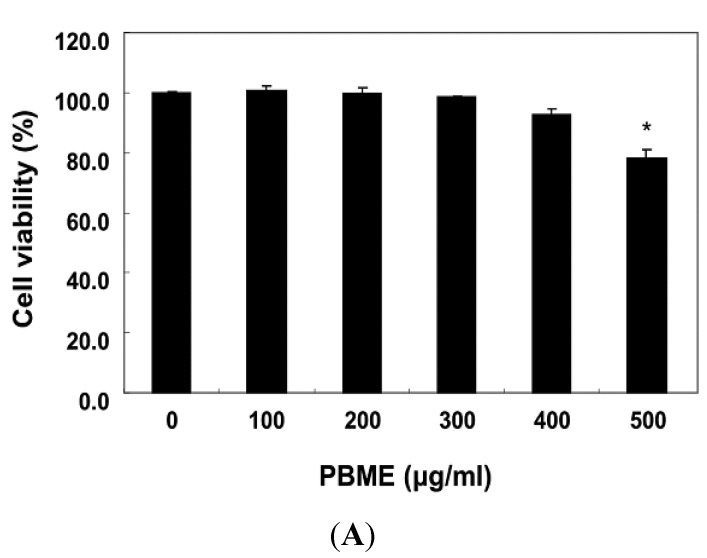

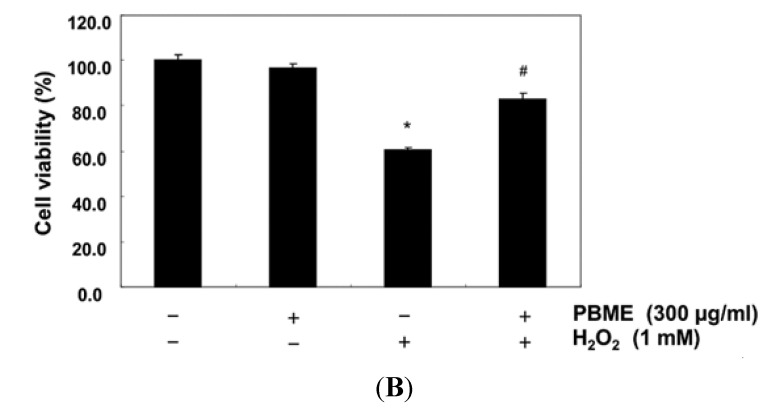
Effects of *P. binghamiae* methanol extract (PBME) on cell viability and H_2_O_2_-induced growth inhibition in C2C12 cells. The cells were treated with various concentrations of PBME for 24 h (**A**) or pretreated with 300 μg/mL of PBME for 1 h and then incubated with and without 1 mM of H_2_O_2_ for 6 h (**B**). The cell viability was assessed with a 3-(4,5-dimethylthiazol-2-yl)-2,5-diphenyltetrazolium bromide (MTT) reduction assay. The results are presented as the mean ± standard deviation (SD) values obtained in three independent experiments (* *p* < 0.05 compared with the control group; ^#^ *p* < 0.05 compared with the H_2_O_2_-treated group).

The cells were first treated with a wide range of PBME concentrations, from 100 to 500 μg/mL, for 24 h to determine the effect of PBME on the viability of C2C12 cells. The PBME treatment up to a concentration of 300 μg/mL did not result in any cytotoxic effects, whereas cell viability dose-dependently decreased at concentrations above 400 μg/mL (Figure 1A). Therefore, 300 μg/mL PBME was chosen as the optimal dose for studying the cytoprotective effect of PBME against the H_2_O_2_-induced cell damage. To examine the protective effect of PBME on H_2_O_2_-induced cytotoxicity, the C2C12 cells were treated with 300 μg/mL of PBME 1 h prior to the H_2_O_2_ treatment, and the cell viability was then measured. Our results indicated that the treatment with 1 mM H_2_O_2_ alone reduced the cell viability by approximately 80% after 6 h. However, the PBME pretreatment significantly protected the cells against the H_2_O_2_-induced reduction in cell viability (Figure 1B), indicating that the exposure of the C2C12 cells to PBME conferred a protective effect against oxidative stress.

### 2.2. PBME Inhibits H_2_O_2_-Induced DNA Damage

**Figure 2:**
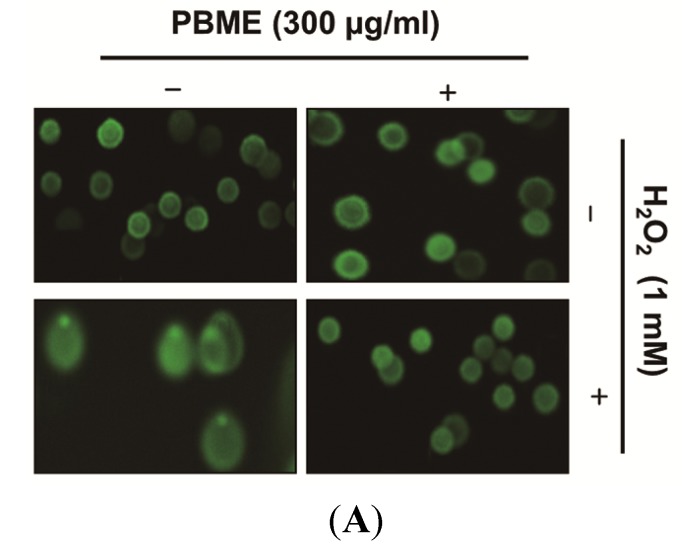

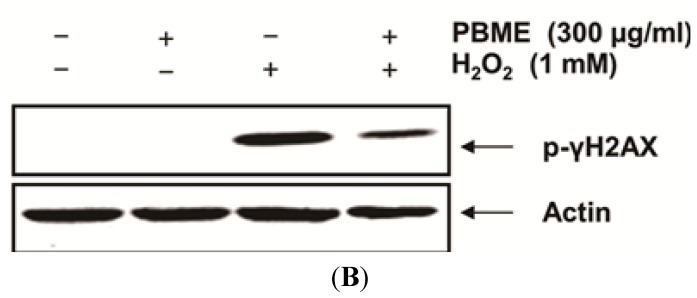
Effect of PBME on H_2_O_2_-induced DNA damage in the C2C12 cells. The C2C12 cells were pretreated with 300 μg/mL of PBME for 1 h and then incubated with and without 1 mM of H_2_O_2_ for 6 h. (**A**) To detect cellular DNA damage, a comet assay was performed, and representative pictures of the comets were taken using a fluorescence microscope (200× original magnification); (**B**) The cells were lysed, and equal amounts of cell lysates were then separated on SDS-polyacrylamide gels and transferred to nitrocellulose membranes. The membranes were probed with specific antibodies against p-γH2A.X and actin, as an internal control, and the proteins were visualized using an enhanced chemiluminescence (ECL) detection system. A representative blot from three independent experiments is shown.

We examined the effects of PBME on H_2_O_2_-mediated damage to C2C12 cell DNA using a comet assay and Western blotting analysis. As shown in Figure 2A, the treatment with H_2_O_2_ alone markedly increased the tail length in the C2C12 cells. However, the PBME markedly reduced this adverse effect. In addition, our results showed that treating the C2C12 cells with H_2_O_2_ resulted in the up-regulation of the level of phosphorylated nuclear histone H2A.X at serine 139 (p-γH2A.X) (Figure 2B). However, the PBME pretreatment decreased the expression level of p-γH2A.X. These suggest that PBME inhibits oxidative stress-induced damage of DNA in C2C12 cells.

### 2.3. PBME Attenuates H_2_O_2_-Induced ROS Accumulation and Apoptosis

**Figure 3:**
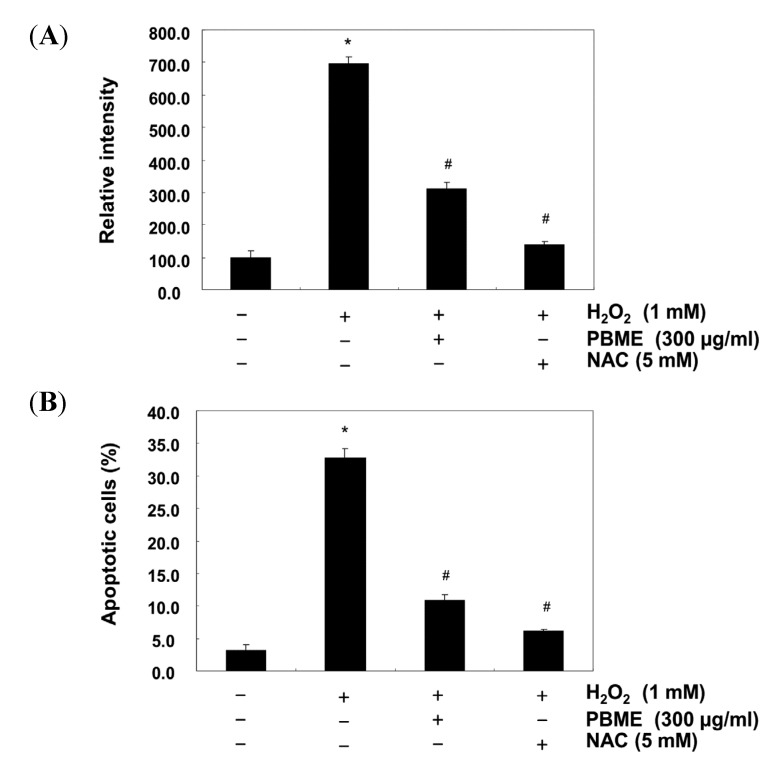
Effect of PBME on H_2_O_2_-induced ROS generation and apoptosis in the C2C12 cells. The C2C12 cells were pretreated with 300 μg/mL of PBME for 1 h and then incubated with and without 1 mM H_2_O_2_ for 6 h. (**A**) To monitor the production of ROS, the cells were incubated at 37 °C in the dark for 20 min with new culture medium containing 10 μM of H2DCFDA. The generation of ROS was measured with a flow cytometer; (**B**) The cells were also stained with annexin V-FITC and propidium iodide (PI), and the percentages of apoptotic cells (annexin V^+^/PI^−^ cells) were then analyzed using flow cytometric analysis. The results are presented as the mean ± SD values obtained in three independent experiments (* *p* < 0.05 compared with the control group; ^#^ *p* < 0.05 compared with the H_2_O_2_-treated group).

We next investigated whether PBME affected intracellular ROS generation by the H_2_O_2_ treatment using a 2′,7′-dichlorodihydrofluorescein diacetate (H2DCFDA) assay. As expected, the ROS levels increased in the H_2_O_2_-treated cells compared with the nontreated cells. However, the levels were significantly inhibited in the presence with PBME (Figure 3A). To further evaluate that the cytoprotective effects of PBME is resulted from the prevention of oxidative stress-induced apoptosis, the frequency of apoptotic cells was detected by flow cytometry. The results showed that the treatment of the cells with PBME prior to H_2_O_2_ exposure strongly protected the C2C12 cells against apoptosis (Figure 3B). As a positive control, the ROS scavenger *N*-acetyl-l-cysteine (NAC) attenuated H_2_O_2_-induced ROS generation, as well as the apoptotic capacity. The results indicate that the H_2_O_2_-induced apoptosis is mediated by ROS generation and that PBME exerts a potent ROS scavenging effect, preventing H_2_O_2_-induced apoptosis.

### 2.4. PBME Upregulates the Expression of HO-1 and Nrf2 Proteins

**Figure 4:**
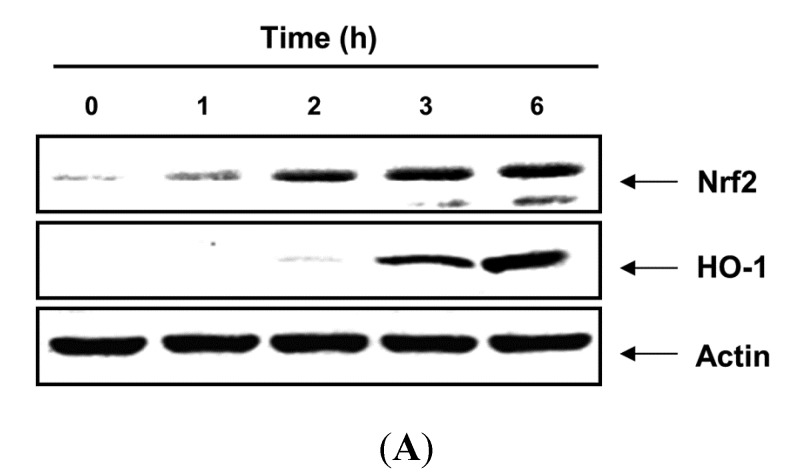

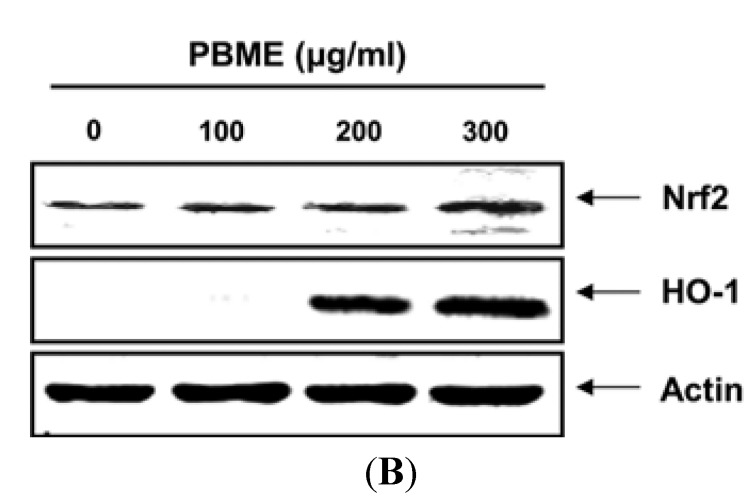
Induction of nuclear factor-erythroid 2 related factor 2 (Nrf2) and heme oxygenase-1 (HO-1) expression by PBME in the C2C12 cells. The cells were incubated with 300 μg/mL of PBME for the indicated periods (**A**) or with various concentrations of PBME for 6 h (**B**). The cellular proteins were separated on SDS-polyacrylamide gels and then transferred onto nitrocellulose membranes. The membranes were probed with the specific antibodies against Nrf2 and HO-1. Actin was used as a loading control.

As it has been well documented that HO-1 is an important component of the cellular defense against oxidative stress, we assessed whether noncytotoxic concentrations of PBME affected HO-1 protein expression. As shown in Figure 4, the treatment of the C2C12 cells with PBME induced the expression of HO-1 proteins compared with the control group in a time- and dose-dependent manner. As several studies have reported that Nrf2 is an important upstream contributor to the mechanism of HO-1 expression [4,5,6], we further examined whether PBME could induce the expression of Nrf2 in C2C12 cells. After exposure to PBME, the Nrf2 levels of the C2C12 cells increased gradually. The increase was strongly correlated with the increase in HO-1 expression (Figure 4).

### 2.5. The Nrf2/HO-1 Pathway Is Involved in the Protection of PBME against H_2_O_2_ Treatment

**Figure 5:**
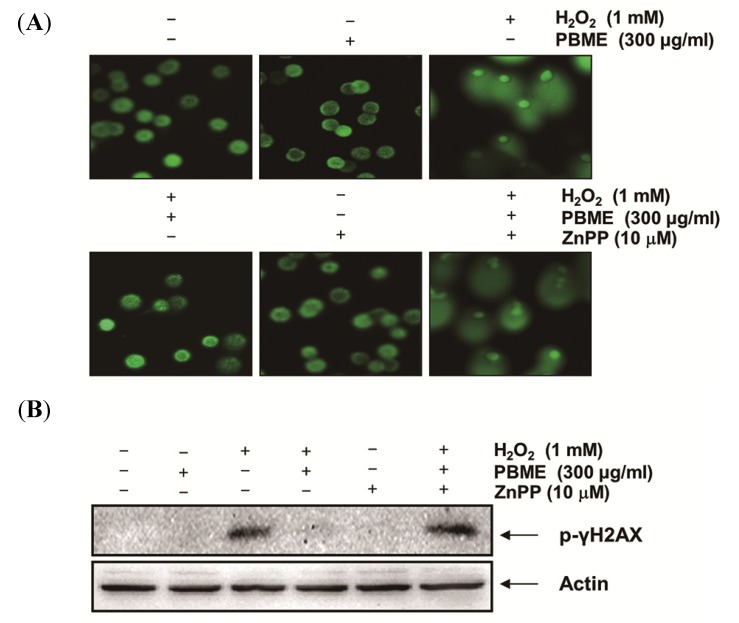
Effects of an inhibitor of HO-1 on PBME-mediated protection of DNA damage by H_2_O_2_ in the C2C12 cells. The cells were pretreated for 1 h with 300 μg/mL of PBME and then treated for 6 h, with or without 1 mM of H_2_O_2_ in the absence or presence of 10 μM of zinc protoporphyrin IX (ZnPP). (**A**) A comet assay was performed, and representative pictures of the comets were taken using a fluorescence microscope at 200× original magnification; (**B**) Cell lysates were prepared and subjected to Western blot analysis with a specific antibody against p-γH2A.X. Actin was used as a loading control.

**Figure 6:**
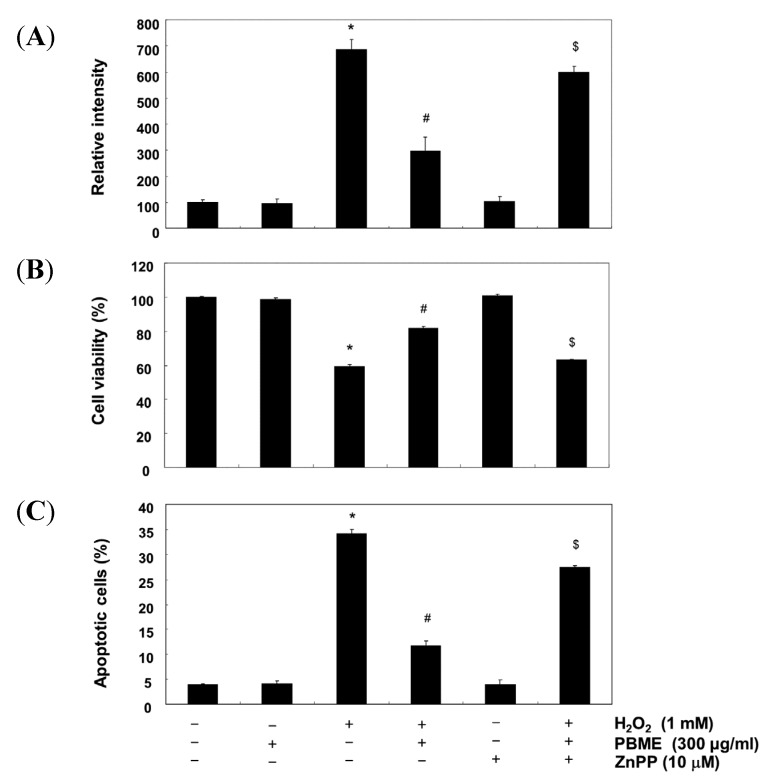
Effects of an inhibitor of HO-1 on PBME-mediated attenuation of ROS formation, apoptosis, and growth inhibition by H_2_O_2_ in the C2C12 cells. (**A**) The cells grown under the same conditions as in Figure 5 and assayed for ROS generation; (**B**) The degree of apoptosis was evaluated using a flow cytometer; (**C**) The cell viability was estimated with an MTT assay. The results are presented as the mean ± SD values obtained in three independent experiments (* *p* < 0.05 compared with the control group; ^#^ *p* < 0.05 compared with the H_2_O_2_-treated group; ^$^ *p* < 0.05 compared with the H_2_O_2_- and PBME-treated group).

To further determine whether the PBME-induced antioxidant and cytoprotective activities against oxidative stress in the C2C12 cells were mediated by activation of the Nrf2/HO-1 pathway, the cells were preincubated with or without a selective inhibitor of HO-1, zinc protoporphyrin IX (ZnPP). As shown in Figure 5, ZnPP inhibited the protective effect of PBME against H_2_O_2_-induced DNA damage. Furthermore, ZnPP abrogated the protective effect of PBME against the H_2_O_2_-induced production of ROS and apoptosis and the reduction of cell viability (Figure 6). These results indicate that PBME exerts its protective effects by inducing the cellular defense mechanism against oxidative stress via the Nrf2-related cytoprotective pathway and that HO-1 plays a crucial role in this protection in C2C12 cells.

### 2.6. Global Discussion

The present study showed that H_2_O_2_ treatment decreased cell viability and induced DNA damage and apoptotic death in the C2C12 cells through ROS generation and the pretreatment with PBME inhibited H_2_O_2_-induced cell death and DNA damage, in addition to the production of ROS. Increased ROS level contributes to mitochondrial dysfunction, which is directly associated with apoptosis [13,14]. Thus, the prevention of ROS accumulation by antioxidant enzymes and detoxifying molecules is crucial for maintaining the balance between oxidants and antioxidants. Therefore, we presumed that PBME might improve mitochondrial function by eliminating the overproduction of ROS induced by H_2_O_2_, thereby reducing the H_2_O_2_-induced apoptosis. To assess the oxidative injury induced by H_2_O_2_, we measured the tail length of DNA and levels of p-γH2A.X, which are widely used markers for the detection of DNA damage [15]. The data from the comet assay and Western blot analysis indicated that the H_2_O_2_ treatment increased the tail length (DNA migration) and the expression of p-γH2A.X, whereas each event was attenuated in the C2C12 cells by treatment with PBME prior to H_2_O_2_ exposure (Figure 2). Therefore, the prevention of apoptosis and DNA damage by PBME might be originated from the powerful antioxidant action of PBME (Figure 3).

Nrf2 is a basic leucine zipper-type transcription factor, which plays essential roles in the induction of detoxifying enzymes, including HO-1. Nrf2 normally exists in cytosol by binding to its cytosolic Kelch-like ECH associated protein-1 (Keap1), which facilitates the ubiquitination and subsequent proteolysis of Nrf2. However, upon exposure to various stresses, Nrf2 dissociates from Keap1 and translocates into the nucleus, whereupon it binds to antioxidant response elements in the promoter regions of activation of distinct sets of genes encoding phase II detoxifying enzymes, as well as several stress-responsive proteins [5,16,17]. Among them, HO-1 is a highly inducible enzyme that catalyzes the rate-limiting step of free heme degradation into Fe^2+^, carbon monoxide, and biliverdin. HO-1 is readily induced in response to oxidative stress, and the induction of HO-1 results in relatively higher resistance to oxidative damage [18,19]. Increasing evidence suggests that the induction of HO-1 expression protects cells against a wide variety of chronic diseases. Therefore, we determined the potential role of HO-1 in H_2_O_2_-induced C2C12 cell damage and PBME-mediated cytoprotection. The data from the Western blot analysis in the present study indicated that PBME induces HO-1 protein expression in a time- and dose-dependent manner, with a concomitant increase in Nrf2 expression (Figure 4). In addition, the inhibition of HO-1 function using an HO-1 inhibitor, ZnPP, effectively abrogated the protective effect of PBME against H_2_O_2_-induced DNA damage (Figure 5). Moreover, the pretreatment with ZnPP significantly attenuated the H_2_O_2_-induced ROS generation, growth inhibition, and apoptosis induction (Figure 6). These results suggest that Nrf2-mediated induction of HO-1 by PBME may, at least in part, participate in the protection against oxidative stress, in agreement with other literature [20,21,22,23,24,25].

## 3. Experimental Section

### 3.1. Preparation of PBME

PBME was purchased from Jeju Bio-Resource Extract Bank (Jeju Technopark, Jeju, Korea). Briefly, fresh *P. binghamiae*, which was collected along the Jeju Island coast of the Republic of Korea in July 2009, was washed three times with tap water to remove salt, epiphytes, and sand from the surface of the samples. The samples were then stored at −20 °C. The frozen samples were lyophilized and homogenized using a grinder before extraction. The dried powder was extracted with 80% methanol (PBME) and evaporated *in vacuo*.

### 3.2. Cell Culture and PBME Treatment

C2C12 myoblasts obtained from the American Type Culture Collection (Manassa, VA, USA) were grown in Dulbecco’s modified Eagle’s medium (DMEM, WelGENE Inc., Daegu, Korea), supplemented with 10% heat-inactivated fetal bovine serum and 100 μg/mL of penicillin/streptomycin antibiotics (WelGENE Inc., Daegu, Korea) in a humidified 5% CO_2_ atmosphere at 37 °C. The PBME was dissolved with a dimethyl sulfoxide (DMSO, Sigma-Aldrich Chemical Co., St Paul, MN, USA) stock solution at a concentration of 50 mg/mL, and the stock solution was then diluted with medium to the desired concentration prior to use.

### 3.3. Cell Viability Assay

As a measure of overall levels of cell viability, the C2C12 cells were assessed in an MTT assay. Briefly, the C2C12 cells were seeded in 6-well plates at a density of 1 × 10^5^ cells per well. After incubation for 24 h, the cells were treated with the indicated concentrations of the PBME in the presence or absence of H_2_O_2_ and/or ZnPP (Sigma-Aldrich Chemical Co., St Paul, MN, USA) for the indicated times. MTT working solution was then added to the culture plates and incubated continuously at 37 °C for 3 h. The culture supernatant was removed from the wells, and DMSO was added to dissolve the formazan crystals. The absorbance of each well was measured at 540 nm with a microplate reader (Molecular Devices, Palo Alto, CA, USA). The effect of the PBME on the inhibition of cell growth was assessed as the percentage of cell viability, where the vehicle-treated cells were considered 100% viable.

### 3.4. Comet Assay (Single-Cell Gel Electrophoresis)

The degree of oxidative DNA damage was assessed in a comet assay. The cell suspension was mixed with 0.5% low melting agarose (LMA) at 37 °C, and the mixture was spread on a fully frosted microscopic slide, precoated with 1% normal melting agarose. After the solidification of the agarose, the slide was covered with 0.5% LMA and then immersed in a lysis solution [2.5 M NaCl, 100 mM Na-ethylenediaminetetraacetic acid (EDTA), 10 mM Tris, 1% Trion X-100, and 10% DMSO, pH 10] for 1 h at 4 °C. The slides were then placed in a gel electrophoresis apparatus containing 300 mM of NaOH and 10 mM of Na-EDTA (pH 13) for 40 min to allow for DNA unwinding and expression of alkali-labile damage. An electrical field was then applied (300 mA, 25 V) for 20 min at 25 °C to draw the negatively charged DNA toward the anode. The slides were washed three times for 5 min at 25 °C in a neutralizing buffer (0.4 M Tris, pH 7.5), followed by staining with 20 µg/mL of PI (Sigma-Aldrich Chemical Co., St Paul, MN, USA). The slides were examined under a fluorescence microscope (Carl Zeiss, Oberkochen, Germany).

### 3.5. Western Blot Analysis

The total cellular proteins were extracted with lysis buffer (20 mM of sucrose, 1 mM of EDTA, 20 μM of Tris-HCl, pH 7.2, 1 mM of dithiothreitol, 10 mM of KCl, 1.5 mM of MgCl_2_ and 5 μg/mL of aprotinin) for 30 min. The protein concentration was measured using a Bio-Rad protein assay (Bio-Rad Lab., Hercules, CA, USA), according to the manufacturer’ instructions. Equal amounts of protein extracts were separated on SDS-polyacrylamide gels and transferred to nitrocellulose membranes (Schleicher & Schuell, Keene, NH, USA). After 2 h blocking with 5% (w/v) nonfat milk in TBST (1.5 M of NaCl, 20 mM of Tris-HCl, 0.05% (v/v) Tween-20, pH 7.4), the membranes were incubated overnight at 4 °C with the desired antibodies. The blots were then washed with TBST 2 h prior to incubation at room temperature with peroxidase-conjugated secondary antibodies (Amersham Co., Arlington Heights, IL, USA). Proteins were visualized with an ECL (Amersham Co., Arlington Heights, IL, USA) detection method, followed by film exposure. The antibodies were purchased from Santa Cruz Biotechnology (Santa Cruz, CA, USA) and Cell Signaling Technology (Danvers, MA, USA).

### 3.6. Measurement of ROS

The intracellular accumulation of ROS was determined using the fluorescent probe, H2DCFDA (Molecular Probes, Eugene, OR, USA). To monitor ROS generation, the cells were incubated with 10 μM of H2DCFDA for 20 min at room temperature in the dark. The ROS production in the cells was monitored with a flow cytometer (Becton Dickinson, San Jose, CA, USA) using Cell-Quest pro software [26].

### 3.7. Assessment of Apoptosis by Flow Cytometry

To assess the induced cell apoptosis rate quantitatively, a fluorescein-conjugated annexin V (annexin V-FITC) staining assay was performed, according to the manufacturer’s protocol (BD Biosciences, San Jose, CA, USA). Briefly, the cells were stained with 5 μL of annexin V-FITC and 5 μL of PI in each sample. After incubation for 15 min at room temperature in the dark, the degree of apoptosis was quantified as a percentage of the annexin V-positive and PI-negative cells by flow cytometry [27].

### 3.8. Statistical Analysis

All measurements were made in triplicate, and all values are presented as the mean ± SD. The results were subjected to an analysis of variance (ANOVA) using the Tukey test to analyze the difference. A value of *p* < 0.05 was considered statistically significant.

## 4. Conclusions

In conclusion, our results demonstrate that PBME effectively suppressed H_2_O_2_-induced oxidative damage by blocking ROS generation. This inhibition may be associated with up-regulation of Nrf2-mediated HO-1, which contributes to a cellular defense mechanism against oxidative stress-induced genotoxic events. Taken together, PBME may have potential as an effective antioxidant, controlling the activity of the Nrf2/HO-1 pathway. Thus, it might be potentially useful therapeutic candidate as an antioxidant agent.
